# Thirty-five Day Fluoxetine Treatment Limits Sensory-Motor Deficit and Biochemical Disorders in a Rat Model of Decompression Sickness

**DOI:** 10.3389/fphys.2017.00604

**Published:** 2017-09-05

**Authors:** Caroline Cosnard, Sébastien De Maistre, Jacques H. Abraini, Laurent Chazalviel, Jean-Eric Blatteau, Jean-Jacques Risso, Nicolas Vallée

**Affiliations:** ^1^Equipe Résidante de Recherche Subaquatique Opérationnelle, Département Environnement Opérationnel, Unité Environnements Extrêmes, Institut de Recherche Biomédicale des Armées, Hopital d'instruction des Armées Sainte-Anne Toulon, France; ^2^Service de Médecine Hyperbare et Expertise Plongée, Hôpital d'Instruction des Armées Toulon, France; ^3^Département d'Anesthésiologie, Université Laval Laval, QC, Canada; ^4^Faculté de Médecine, Université de Caen Normandie (UNICAEN) Caen, France; ^5^Université de Toulon La Garde, France

**Keywords:** mitochondrial DNA, venous gas emboli, oxidative stress, oxygen, depression, capillary leak

## Abstract

According to the OECD statistical base for 2014, anti-depressants will, on average, be distributed at a rate of 62 daily doses per 1,000 inhabitants for the 25 countries surveyed (Health at a glance: Europe 2014; OECD Health Statistics; World Health Organization and OECD Health Statistics, 2014). Divers must be concerned. On another hand, divers are potentially exposed to decompression sickness including coagulation inflammation and ischemia, which can result in neurological lesions or even death. The purpose of this study is to assess whether chronic treatment with anti-depressants may represent a contraindication to the practice of an at-risk activity, such as, scuba diving, or even presents a benefit by attenuating the severity of the symptoms. We study for the first time the effect of a 35-day fluoxetine treatment (20 mg/kg) on the occurrence of decompression sickness in laboratory rats (*n* = 79). Following exposure to the hazardous protocol, there is a significant correlation between the type of treatment and the clinical status of the rats in favor of a better clinical prognosis for the rats treated with fluoxetine with a significantly higher number of No DCS status and a lower number of Severe DCS status in the Flux, compared to Controls. The treatment modifies the rat performances both significantly and favorably during the physical and behavioral tests, just like their biological and biochemical constants. After decompression, rats under treatment display lower sensory-motor deficit and lowers biochemical disorders. From a biological point of view, we conclude fluoxetine should not be seen as a contraindication for diving on the basis of anticipated increased physiological risk.

## Introduction

Gas embolism following an at-risk decompression induces disseminated coagulation, systemic inflammation, and ischemia, which can cause neurological disorders or even death.

In animals, we have been able to demonstrate (Blatteau et al., 2012, 2015; Vallee et al., 2016) that fluoxetine administered at a high dose before hyperbaric exposure presents a curative interest in the event of the occurrence of decompression sickness. Several hypotheses could explain the beneficial effect of fluoxetine on DCS. Firstly, fluoxetine administered in a single high dose (50 mg/kg) has anti-inflammatory properties, as is shown by a reduction in the circulating level of inflammatory markers (Branco-De-Almeida et al., 2011; Blatteau et al., 2012, 2015). A reduction in infiltration of neutrophils and preservation of the number of platelets and red blood cells are also observed (Blatteau et al., 2012). CVA and DCS are accidents which both include ischemia. The anti-inflammatory action of fluoxetine in both cases could counter the ischemic process. Secondly, fluoxetine has been proved to be effective in post-CVA recovery (Chollet et al., 2011) due to activation of the mTOR signaling pathway, which plays an important role in cell growth and proliferation and control of synthesis of the proteins required for synaptogenesis and dendritogenesis. Thirdly, the inhibition of NMDA (NR2A) channels by fluoxetine limits the glutamatergic excitotoxicity induced by non-apoptotic cell death. This non-voltage dependent inhibition could therefore be neuroprotective (Szasz et al., 2007). Finally, the inhibition of SERT transporters, which are present on the neuronal membranes, increases the serotonin level in the synaptic cleft, which is the source of its action as an anti-depressant (Branco-De-Almeida et al., 2011), but they are also present on platelet membranes and have an anti-coagulant effect (Halperin and Reber, 2007). Blood thinning by SSRIs would therefore be beneficial in limiting prothrombotic processes. Conversely, a decompression accident affecting the brain or the spinal cord could be aggravated by a hemorrhagic transformation. Administration of fluoxetine also has the effect of dilating the small cerebral (Ungvari et al., 1999; Sanchez-Ortiz et al., 2007), and cutaneous (Lin, 1978) arteries as has been demonstrated in the rat.

Other arguments seem to indicate that fluoxetine would be an aggravating factor in DCS. Initially the inhibition of the TREK 1 channel causes dissociation of the C-terminal domain from the cell membrane and prevents the passage of potassium ions (Chen et al., 2007). This counters the reduction in the neuroprotective cell excitability exercised by TREK 1. Our previous studies, which blocked the TREK 1 channel, either by using native KO mice (Vallee et al., 2012), or by internalizing the channel pharmacologically with spadin (Vallee et al., 2016), to have “anti-depressed” mice models, have highlighted increased susceptibility to decompression sickness with an unfavorable prognosis for survival (Vallee et al., 2012, 2016). Then, Baek et al. (2015) observed that in mice treated for more than 14 days, fluoxetine increased inflammatory intestinal problems, contrary to the anti-inflammatory action observed during a single acute dose. Finally, fluoxetine also has hepatotoxic side effects because it is metabolized in the liver by demethylation into norfluoxetine (Johnson et al., 2007).

Also, it was important to respond to the toxicity of a chronic treatment by fluoxetine on the risk of DCS. In fact, fluoxetine, an SSRI (selective serotonin reuptake inhibitor) initially distributed under the brand, Prozac (Lilly France S.A.S.), is a chronically prescribed anti-depressant. Depression, affecting 3–5% of the global population (Lepine and Briley, 2011) raises a very serious question: is taking an anti-depressant a contraindication to practicing an at-risk activity such as, scuba diving? Depression is not as such a contraindication to the practice of scuba diving, even if this activity requires good psychic stability given the risks it carries. However, we will not focus on the attentiveness aspect this sport requires for monitoring parameters such as, depth control or adherence to decompression time. Conversely this work concerns the effect of anti-depressant medication in the context of decompression sickness. This study is the first to assess the somatic effect of a chronic treatment with fluoxetine at the normal doses on the occurrence of decompression sickness in laboratory rats.

The purpose of this study is to assess whether chronic treatment with anti-depressants may represent a contraindication to the practice of an at-risk activity, such as, scuba diving, or even presents a benefit by attenuating the severity of the symptoms. On a rat model treated with fluoxetine for 35 days, we measured the clinical and biochemical effects of hyperbaric exposure causing DCS.

## Materials and methods

**Figure 1:**
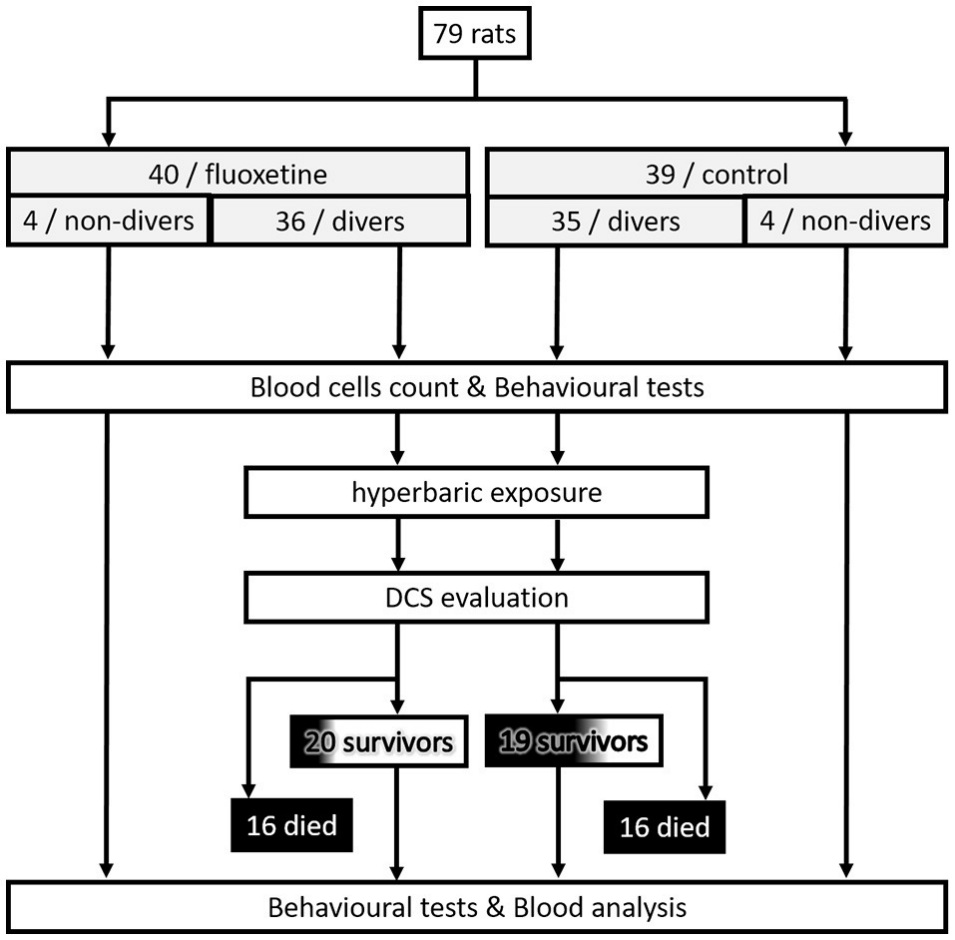
Flowchart describing the experimental design.

The experimental design can be follow in Figure 1.

### Animals and ethical statement

All procedures involving experimental animals were in line with European Union rules (Directive 2010/63/EU) and French law (Decree 2013/118). The Ethics Committee of the Institut de Recherche Biomédicale des Armées approved this study. According to our Animal Care Committee, a scoring system inspired by Swiss veterinary guidelines was implemented to ensure the welfare of animals. For each animal, a dedicated observer scored the stress or pain (from 0 to 3) relating to specific criteria listed on a form (see Supplementary Material for more information). Degree 3 pain (very painful) in one case or a total score of 12 in the table were the ethical endpoints. On this sheet, the most commonly found were: vocalizing, aggression or withdrawn behavior, reduction in exploratory behavior, licking, closed eyes, tears, bubbles in the eyes, high respiratory rate, runny nose, fur bristling, labored breathing, convulsions, paralysis, difficulty moving, and problems with the fore or hind limbs (classified as motor disorders). In this study, no score reached 12 and there was no need to cull the animal based on these criteria. Actually, animals displaying Degree 3 convulsions died very rapidly. At the end of the experiment, rats were anesthetized first with halothane (5% in oxygen, Halothane, Belamont, France) in order to gain time and to minimize stress, and then with an intraperitoneal injection of a mixture of 16 mg/kg xylazine (Rompum® 2%, Bayer Pharma) and 100 mg/kg ketamine (Imalgène® 1000, Laboratoire Rhône). Our investigator (NV) is associated with Agreement Number 83.6 delivered by the Health and Safety Directorate of our department, as stated in the French rules R.214-93, R-214-99, and R.214-102.

Rats were housed in an accredited animal care facility. Rats were kept in group cages (two per cage) both during rest and during the experiments and maintained on a regular day (6:00 am–6:00 pm)/night (12 h) cycle. Food (AO3, UAR) and water were provided ad libitum and the temperature was kept at 22 ± 1°C.

The rats were separated into two groups: 39 control rats (Ctrl) and 40 rats (Flux) treated with an anti-depressant daily. The Flux rats drank water supplemented with fluoxetine (Fluoxetine Zentiva, Laboratoires Sanofi, 20 mg dispersible tablets) for 36 days. The tablets were diluted so as to correspond to a dose of 20 mg/kg of fluoxetine. The dose was regularly adjusted depending on the volume of water drunk and the weight of the rats. Drinking bottles were weighed every day to control hydration, and changed every 2 days. The rats were all weighed once a week to check their weight gain. They were 12 weeks old at the time of the hyperbaric protocol.

### Hyperbaric exposure

Samples of eight rats (four per cage) from the flux pool and the ctrl pool were subjected to the hyperbaric protocol in a 200-l tank fitted with three observation ports. The rats were free to move around the cage.

The compression protocol involved two ramps of pressure increase, first at 0.1 atm/min up to 1 atm, followed by 1 atm/min up to 9 atm; 9 atm corresponds to the pressure where animals were kept for 45 min before decompression. The decompression rate was 60 atm/min up to the surface. Compression and decompression were automatically controlled by a computer linked to an analog/digital converter (NIUSB-6211, National Instrument, USA) with two solenoid valves (Belino LR24A-SR, Switzerland) and a pressure transmitter (Pressure Transmitter 8314, Burket Fluid Control System, Germany). The software was programmed on a DasyLab (DasyLab National Instrument, USA) by our engineer. The software also controlled the temperature and oxygen rate. Compressed air was generated using a diving compressor (Mini Verticus III, Bauer Comp, Germany) coupled to a 100-l tank at 300 bars. The oxygen analyzer was based on a MicroFuel electrochemical cell (G18007 Teledyne Electronic Technologies/Analytical Instruments, USA). The temperature inside the tank was monitored using a platinum resistance temperature probe (Pt 100, Eurotherm, France).

Water vapor and CO2 produced by the animals were captured with soda lime (<300 ppm captured by the soda lime) and seccagel (relative humidity: 40–60%). Gases were mixed by an electric fan. The day–night cycle was respected throughout.

### Behavior and clinical observations

At the end of decompression, the rats were first observed over 30 min in an open field. The possible occurrence and the time to onset of the following manifestations were recorded: respiratory distress, moving difficulties, convulsions, and death. Specific motor sensory tests were performed 30 min after the hyperbaric exposure. Beam walk, righting reflex, dynamic weight-bearing (DWB) assessment, and thermal stimulation tests were performed within 2 h of the dive. Beam Walk and DWB tests were also performed 2 weeks before the dive.

The beam walk performance was tested using seven wooden planks of different widths (7.7–1.7 cm), which were 1.5 m long and elevated 110 cm above the floor. Rats were placed on the widest plank and the ability to cross the plank without the feet slipping in two trials was observed. The procedure was repeated on successively narrower planks. The narrowest plank that a rat could cross without slipping was recorded i.e., No.7 for 1.7 cm, No.6 for 2.7 cm, No.5 for 3.7 cm, No.4 for 4.7 cm, No.3 for 5.7 cm, No.2 for 6.7 cm, and No.1 for 7.7 cm.

The righting reflex was elicited by holding the rat in one hand and turning it over on its back 7 or 8 cm above a covered table surface. The way the animal tried to regain its original position with its feet down was studied. To avoid injuring the rats when testing for righting, the animals with a motor score below 3 were tested for their ability to turn by dropping them from a height of 2 or 3 cm. Righting: 0 No attempt to right itself, 1 Weak or delayed attempt to right or rights itself in the direction of the roll, 2 Normal righting counter to the direction of roll.

The DWB distribution was assessed by a biometric floor instrumented cage (DWB, Bioseb Development, Vitrolles, France). The device consisted of a Plexiglas box (width 22 × length 22 × height 30 cm) with a calibrated weight transducer pad composed of 44 × 44 captors (TEKSCAN, Boston, MA). The rat was allowed to move freely within the box for 4 min each. Using a synchronized video recording and a scaled map of the stimulated captors, each of the rat's paws was validated by an observer and identified as a unique paw. The pressure exerted by each paw (in grams) was only measured when the four paws were in contact with the biometric floor and then normalized by the total weight of the rat. Ratios distinguishing the forepaws vs. hind paws and the right vs. left side were calculated to assess the weight-bearing distribution: (1) the sum of the right and the left forepaws (F) was normalized by the sum of the right and the left hind paws (H) (F/H ratio); (2) the left forepaw (LF) was normalized by the right forepaw (RF) (LF/RF ratio); (3) the left hind paw (LH) was normalized by the right hind paw (RH) (LH/RH ratio). The time spent on three paws and on four paws (in s) was also measured to determine the solicitation of the paws in postural stability. The time period spent on four paws was normalized by the time period spent on three paws (4P/3P ratio).

The thermal stimulus was delivered using the Hargreaves technique (7371 Plantar Test from Ugo Basile S.R.L. Biological Research Apparatus, Comerio, Italia). Rats were placed in a clear Plexiglas box resting on an elevated glass plate. Following acclimatization, a radiant beam of light at 60°C was positioned under the hind paw, and the average time for the rat to remove the paw from the thermal stimulus over three trials was electronically recorded in seconds as the paw withdrawal latency (PWL). The intensity of the beam was set to produce basal PWL's of ~8–10 s. A maximal PWL of 25 s was used to prevent excessive tissue damage due to repeated application of the thermal stimulus.

### Anesthesia and sacrifice

All rats were anesthetized 120 min after surfacing (after behavioral and clinical tests) first with isoflurane 4% (Isoflo, Axience SAS, France) to minimize stress, and then by intraperitoneal injection of a mixture of 16 mg/kg xylazine (Rompum® 2%, Bayer Pharma, Germany), 100 mg/kg ketamine (Imalgene®1000, Rhône laboratory, France) and 1.65 mg/kg of acepromazine (Calmivet®, Vétoquinol, France).

Rats were kept for aortic blood sampling for biochemistry and then sacrificed by injecting pentobarbital (200 mg/kg ip, Sanofi Santé, France).

### Full blood analysis

Blood cells counts were analyzed in 15 μL samples taken from the tip of the tail and diluted in the same volume of 2 mM EDTA (Sigma, France). Blood tests were carried out using an automatic analyzer (ABCvet, SCIL Animal Care Company, France) on samples taken 30 min before the dive and again 120 min afterwards. The second test values were corrected according to the hematocrit variation.

Under general anesthesia and 2 h after the end of the hyperbaric protocol, blood samples were collected from the abdominal aorta with a blood collection set (Vacutainer Brand, Becton Dickinson, Meylan, France) for biochemistry, cytokines, and circulating oligonucleotides analysis.

Blood biochemistry [Na^+^, K^+^, Ca^2+^, creatinine kinase, glucose, blood urea nitrogen (BUN), creatinine, transaminase, bilirubin, albumin, globulin, total proteins, lactate, cholesterol, triglycerides] was conducted with automatic analyzers (Vetscan VS2, Abaxis Veterinary Company, France; Reflovet Plus SCIL Animal Care Company, France; and Accutrend Plus, Roche Diagnostic, USA) on lithium heparin (4 mL, Lithium Heparin 68 I.U., BD Vacutainer, Becton Dickinson, Plymouth, UK) blood samples. Hemolytic samples were rejected.

### Circulating oligonucleotides

Circulating oligonucleotides were quantified from blood samples (EDTA tubes: 2 mL, K2E 3,6 mg, BD Vacutainer, Becton Dickinson, Plymouth, UK). 3 detection processes were performed.

The levels of circulating oligonucleotides in the blood plasma were measured (1) in accordance with the method previously used in the laboratory (Vallee et al., 2013) including double centrifuging before the extraction/purification phase, which was followed by a qPCR targeting a mitochondrial locus (marked mDNA), and which includes (2) the measurement by spectrophotometry post-extraction usually used to adjust the parameters of the qPCR, and (3) by direct measurement at the different stages (centrifuging/extraction/amplification) using a fluorochrome targeting the double strands of DNA without distinction.

Quantification of oligonucleotides as previously performed (Vallee et al., 2013) in blood, required immediate centrifuging for 10 min at 1,500 g at 4°C, and a second one of supernatants for 10 min at 20,000 g at 4°C. The samples collected were stored at −80°C until analysis.

Five microliters of synthetic DNA (Yakima Yellow-BHQ-1™ probe, Eurogentec, Seraing, Belgium), for internal normalization, was added to 140 μL plasma samples before extraction. DNA was extracted using a QIAcube automat (Qiagen, Venlo, Nederland) and the NucleoSpin RNA Virus kit (Macherey-Nagel, Düren, Germany), according to the manufacturer's instructions.

Quantitative PCR was carried out with a LightCycler 480 II (LightCycler 480 Software Release; Roche Diagnostics, Mannheim, Germany) on 2 μL DNA extract added to 8 μL reaction mixture. For the negative controls, water was substituted for the extract. The reaction mixture for mitochondrial DNA amplification contained 1 μL water, 1 μL primers F and R (1 μM; Eurogentec, Seraing, Belgium), and 5 μL of an amplification mixture SyberGreen (Go Taq qPCR Master Mix 2X, Promega, Madison, USA), mitochondrial primers [mitochondrial primer forward (F) (5′ ACC TCG ATG TTG GAT CAG 3′); mitochondrial primer reverse (R) (5′ TAG ATA GAA ACC GAC CTG G 3′)]. The reaction mixture for internal control amplification contained 2 μL water, 1 μL primers delivered by the manufacturer (Eurogentec, Seraing, Belgium), and 5 μl of an amplification mixture SyberGreen (Go Taq qPCR Master Mix 2X, Promega, Madison, USA). Thermocycler settings were: initialization step 94°C/10 min (denaturation step 94°C/15 s; annealing 55°C/20 s; and elongation step 72°C/35 s) × 45 cycles; pre-melting 95°C/10 s, melting 55°C/10 s then up to 95°C, cooling 37°C/30 s. All assays were carried out in duplicate. Specificity was checked by melting curve analysis, as described previously.

DNA double strand fluorometric measurements were performed on samples after both centrifugation and extraction. Four microliters of superrnatant or extract was mixed with 196 μL of a reaction mixture (QuantiFluor ONE dsDNA Dye, Promega, Madison, USA), incubated according to the manufacturer's instruction, and measured with Quantus Fluorometer (Promega, Madison, USA).

Spectrophotometric quantification from 1.5 μL DNA extract was performed after extraction using Nanodrop-1000 and its software (Thermo Scientific, Wilmington, USA).

### Clinical status

The Lethal DCS status includes rats which died in the 2 h following the end of the hyperbaric protocol. The Severe DCS status was attributed when the rat presented severe locomotor signs in the form of paresia or paralysis of at least 1 limb and/or a beam walk test score reduced by at least two points. The DCS status includes rats without serious motor symptoms, with high transaminase values (±10% of extreme values for the controls) suggesting the presence of bubbles in the liver (L'abbate et al., 2010; Vallee et al., 2016). The other rats were considered as No DCS.

### Statistical analyses

Individual blood cell count data were calculated as the percentage change from baseline (the measurement before hyperbaric exposure). Numerical data points are expressed as mean and standard deviation. A contingency table was used for independence and association tests coupled with the χ^2^ significance test. Different groups were compared using the Mann-Whitney (MW) test and matched comparisons within groups were analyzed using the Wilcoxon (W) test. Multiple comparisons were performed using the Kruskal-Wallis test followed by the Bonferroni-Dunn *post-hoc* test. The Kolmogarov-Smirnov test was used to compare the distribution of water consumption over the time of treatment. The significance threshold was 5% and *p*-values below were assumed to indicate significant differences.

## Results

### Monitoring of the animal population

Over the 36 days of treatment, the Flux rats drank more than the Ctrl rats in terms of distribution with a lower consumption in the first 5 days and then a higher consumption from the 10th day of treatment (Kolmogorov–Smirnov_Ctrl/Flux_, *n* = 39/40, *p* < 0.0001) and also in terms of average quantity per day (Beverage: Ctrl = 33.1 ± 1.5 mL, Flux = 35.2 ± 3.7 mL; MW_Ctrl/Flux_, *n* = 39/40, *p* < 0.0001).

The weights of the rats subjected to the hyperbaric protocol were similar (Weight: Ctrl = 348.3 ± 19.2 g, Flux = 340.1 ± 22.4 g; MW_Ctrl/Flux_, *n* = 35/36, *p* = 0.099). Weight had no incidence on the clinical data (KW_SevereDCS/DCS/noDCS/Lethal_, *n* = 13/6/20/32; *p* = 0.151).

### Clinical status of the rats following the dive

**Figure 2:**
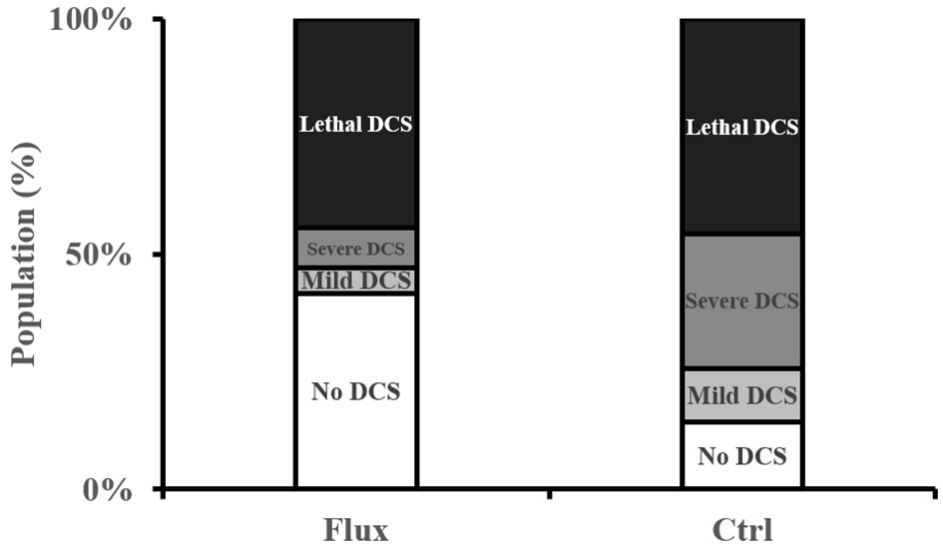
Clinical status after the hyperbaric protocol exposure.

In the control rats exposed to the hyperbaric protocol (Figure 2), there was 45.7% mortality at 2 h (Lethal DCS), 28.6% of rats presented symptoms of severe but not lethal DCS (Severe DCS), 11.4% moderate DCS, and 14.2% of rats No DCS. In the rats treated with fluoxetine, 44.4% of animals died, 8.3% were classified Severe DCS, 5.5% DCS, and 41.6% No DCS.

There is a significant correlation between the type of treatment and the clinical status of the rats (contingency table $\chi_{\text{Ctrl}/\text{Flux}}^{2}$ = 9.424, *n* = 35/36, *p* = 0.021), in favor of a better clinical prognosis for the rats treated with fluoxetine with a significantly lower number of No DCS status in the Ctrl and a significantly lower number of Severe DCS status in the Flux.

### Clinical and behavioral analyses

**Figure 3:**
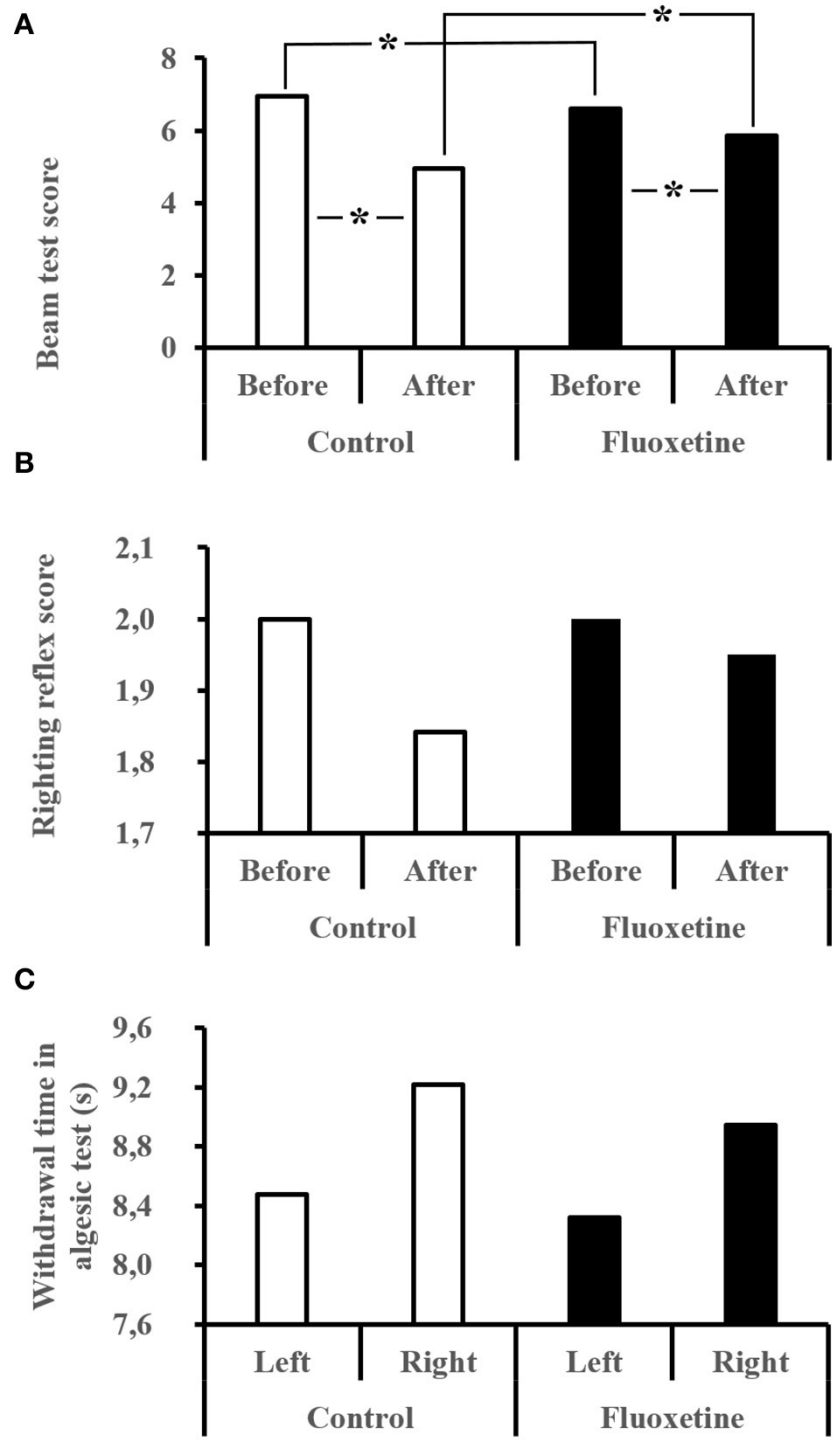
**(A–C)** Behavioral performances after the hyperbaric exposure. **(A)** Performance for the beam test. The best score is 1 and the worst is 7. **(B)** Righting reflex. The best score is 2 and the worst is 0. **(C)** Algesia test performed with infrared stimulation on left and right hind paws. Average time for the rat to remove the paw from the thermal stimulus. *Significant difference (*p* < 0.05).

Before the dive the Flux rats experienced slightly more difficulties of significance on moving on the beam (Figure 3A) above the void (Beam: Ctrl = 6.88 ± 0.32 vs. Flux = 6.55 ± 0.50; MW_Ctrl/Flux_, *n* = 35/36, *p* = 0.0002). Following the dive, the performance of Ctrl and Flux survivors had deteriorated with a global locomotor impairment (Beam: Ctrl = 4.94 ± 2.00 vs. Flux = 5.85 ± 1.18; Ctrl, W_before/after_, *n* = 19/19, *p* < 0.0001; Flux, W_before/after_, *n* = 20/20, *p* = 0.039) more severe in the Ctrls (delta Beam: Ctrl = 2.00 ± 2.05 vs. Flux = 0.75 ± 0.37; MW_Ctrl/Flux_, *n* = 19/20, *p* = 0.039). As expected, the levels of performance deterioration were higher in the Severe DCS rats than in the DCS and No DCS and No Divers rats, all treatments included (delta Beam: Severe DCS = 3.46 ± 1.61, DCS = 0.50 ± 0.54, No DCS = 0.25 ± 0.64, No Divers = 0.37 ± 0.74: KW_SevereDCS/DCS/NoDCS/NoDivers_, *n* = 13/6/20/8, *p* < 0.0001).

The performance deterioration levels for the righting reflex test (Figure 3B) are higher in Severe DCS rats than in No DCS rats, all treatments included (delta reflex: No DCS = 0.00 ± 0.00 vs. Severe DCS = 0.30 ± 0.630: KW_SevereDCS/DCS/noDCS/NoDivers_, *n* = 13/6/20/8; *p* = 0.042), without there being any difference related to the treatment.

For the infrared stimulation (Figure 3C) in the palm of the hind paw, reduced algesic sensitivity in the No Divers Ctrl rats was observed compared to the No Divers Flux rats only for the left paw (InfraRed stimulation on left paw; L Paw No Divers: Ctrl = 12.22 ± 3.78 s vs. Flux = 5.97 ± 1.61 sec; KW_Ctrl/NoDiversCtrl/Flux/NoDiversFlux_, *n* = 19/4/20/4; *p* = 0.040;), but without significant lateralization in non-exposed rats (No divers MW_CtrlR/L_, *n* = 4/4, *p* = 0.312; MW_FluxR/L_, *n* = 4/4, *p* = 0.191). This difference did not appear in rats that had dived, whatever the treatment or symptoms (Left Paw: Ctrl KW_NoDivers/SevereDCS/DCS/NoDCS_, *n* = 4/10/4/5, *p* = 0.208, Flux KW_NoDivers/SevereDCS/DCS/NoDCS_, *n* = 4/3/2/15, *p* = 0.258; Right Paw: Ctrl KW_NoDivers/SevereDCS/DCS/NoDCS_, *n* = 4/10/4/5, *p* = 0.930, Flux KW_NoDivers/SevereDCS/DCS/NoDCS_, *n* = 4/3/2/15, *p* = 0.137). Globally, there is no greater difference between the no diver rats and the divers (Left Paw MW_CtrlDivers/NoDivers_, *n* = 19/4, *p* = 0.080; Left Paw MW_FluxDivers/NoDivers_, *n* = 20/4, *p* = 0.121; Right Paw MW_CtrlDivers/NoDivers_, *n* = 19/4, *p* = 0.598; Right Paw MW_FluxDivers/NoDivers_, *n* = 20/4, *p* = 0.510).

**Figure 4:**
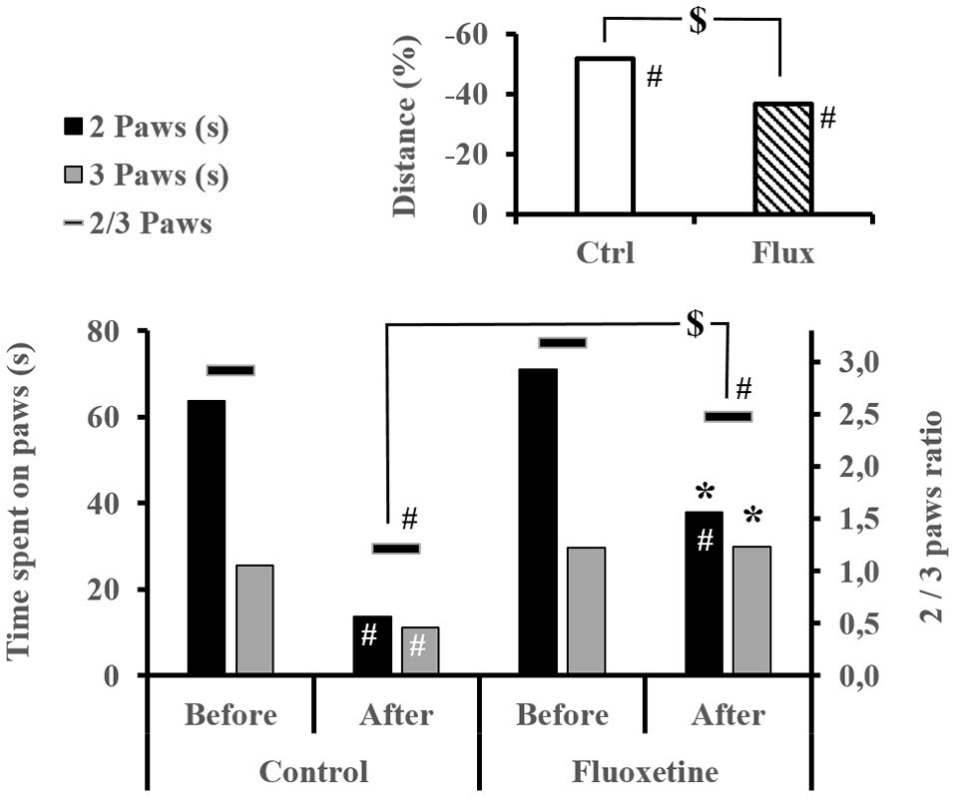
Exploratory behavior by video tracking. Time spent on 2 or 3 paws and its ratio, and the mean distance walk in each group, within the video box for 4 min. ^#^Intragroup significant difference (*p* < 0.05) between before and after the dive. ^*^Significant difference (*p* < 0.05) between groups after the dive for the evolution of the same parameter. ^$^Trend (*p* < 0.01) at the intergroup level.

Before the dive, the treatment did not influence exploratory behavior (Figure 4), in terms of time passed on 2 or 3 paws (2 paws KW_Ctrl/Flux_, *n* = 39/40, *p* = 0.247; 3 paws KW_Ctrl/Flux_, *n* = 39/40, *p* = 0.499; ratio 2/3 Paws KW_Ctrl/Flux_, *n* = 39/40, *p* = 0.590) or, in terms of distance covered (distance KW_Ctrl/Flux_, *n* = 39/40, *p* = 0.499). Performance were significantly deteriorated after the dive, except for the time spent on 3 paws in the flux pool (Figure 4). Globally the Severe DCS rats have inhibited exploratory behavior in comparison to the No DCS (2 paws KW_SevereDCS/DCS/NoDCS_, *n* = 13/6/20, *p* = 0.001; ratio 2/3 paws KW_SevereDCS/DCS/NoDCS_, *n* = 13/6/20, *p* < 0.0001). At intragroup level, this same difference is only found in non-treated rats (Ctrl 2 paws KW_NoDivers/SevereDCS/DCS/NoDCS_, *n* = 4/10/4/5, *p* = 0.011; Ctrl ratio 2/3 paws KW_NoDivers/SevereDCS/DCS/NoDCS_, *n* = 4/10/4/5, *p* = 0.006). During video tracking after the dive, the surviving Flux rats explored their space more significantly than the surviving Ctrl rats because they spent more time on two or three paws with a tendency to travel further (2 paws KW_Ctrl/Flux_, *n* = 19/20, *p* = 0.014; 3 paws KW_Ctrl/Flux_, *n* = 19/20, *p* = 0.005; ratio 2/3 Paws KW_Ctrl/Flux_, *n* = 19/20, *p* = 0.096; distance KW_Ctrl/Flux_, *n* = 19/20, *p* = 0.068).

### Full blood count

Before the dive a mild effect from the chronic treatment with fluoxetine may be seen. Also it appears that the Flux animals have increased Mean Corpuscular Volume (KW_MCV_, *n* = 39/40, *p* = 0.029) and Mean Corpuscular Hemoglobin (KW_MCH_, *n* = 39/40, *p* = 0.030), which could be linked to a liver disorder (Blann and Ahmed, 2014). Furthermore, no significant rheological predisposition has been observed (in terms of prior Full Blood Count) related to the occurrence of an accident with hindsight, particularly concerning the hematocrit/hydration.

**Figure 5:**
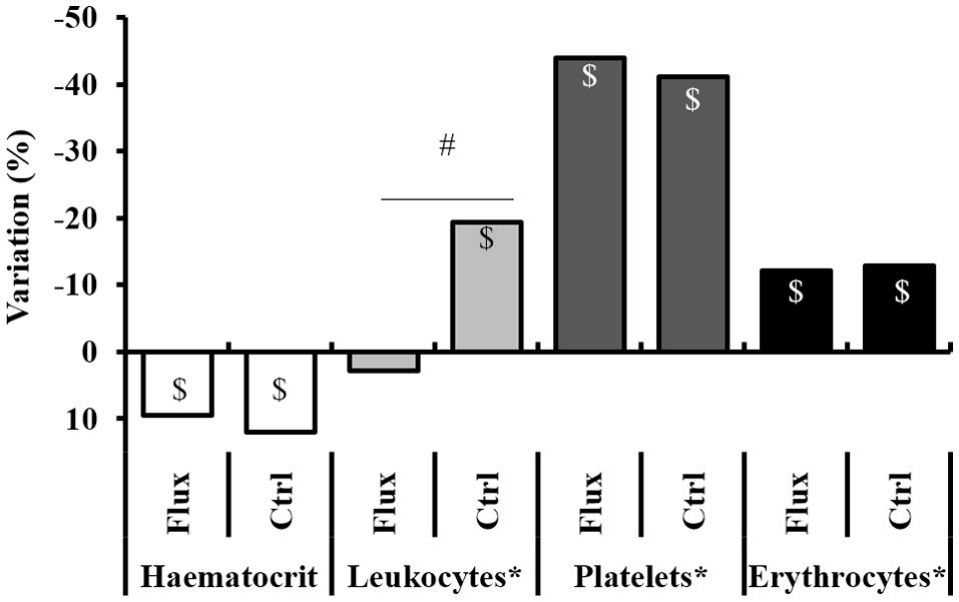
Full blood count variation, between before and after the hyperbaric exposure. Ctrl, control; Flux, Fluoxetine; ND, non-diver. ^*^Corrected according to the individual hematocrit variation. ^$^Significant difference (*p* < 0.05) between before and after the dive for the same parameter. ^#^Significant difference (*p* < 0.05) between groups after the dive for the evolution of the same parameter.

The second sampling carried out after the hyperbaric exposure showed rheological changes (Figure 5 and Table 1).

**Table 1:** Full blood count variation after hyperbaric exposure.

***p*-Values for before/after comparisons (Wilcoxon test)**	**Ctrl**		**Flux**		**Ctrl ND**		**Flux ND**	
	***n*** = **19**		***n*** = **20**		***n*** = **4**		***n*** = **4**	
Hematocrit	<**0.0001**	↑	<**0.0001**	↑	**0.029**	↑	**0.029**	↑
Leucocytes^*^	**0.003**	↓	0.0867	↔	0.100	↔	0.100	↔
Platelets^*^	<**0.0001**	↓	<**0.0001**	↓	**0.029**	↓	**0.029**	↓
Erythrocytes^*^	<**0.0001**	↓	<**0.0001**	↓	0.100	↔	0.100	↔
MCH	0.066	↔	**0.0004**	↓	0.146	↔	0.559	↔
MCV	<**0.0001**	↑	<**0.0001**	↑	**0.027**	↑	**0.026**	↑

* *Corrected according to the individual hematocrit variation*.

*Ctrl, control; Flux, Fluoxetine; ND, non-diver*.

*Bold numbers note significant variations*.

After the dive an increased hematocrit was observed, i.e., a hemoconcentration in all the animals, which has involved correcting the count values (marked ^*^) for each individual. A significant difference was revealed between the raw values related to clinical status with a higher hemoconcentration in the Severe DCS compared to the No DCS (HCT: No DCS = 55.5 ± 13.7%; Severe DCS = 67.0 ± 13.4%; KW_NoDivers/SevereDCS/DCS/NoDCS_, *n* = 8/13/6/20, *p* = 0.047), which seems to support a threshold value of severe pathology in these precise experimental conditions. However, there is no significant difference in the proportion of this variation which can be directly attributed to the treatment (KW_Ctrl/Flux/CtrlNoDivers/FluxNoDivers_, *n* = 19/20/4/4, *p* = 0.593) or the clinical data (KW_NoDivers/SevereDCS/DCS/NoDCS_, *n* = 8/13/6/20, *p* = 0.145), which suggests that the variation in the hematocrit is potentially due to the stress of the second sample collection or the hyperbaric protocol (simulated or real) on the one hand, and also to the effects of the hyperbaric exposure on the other hand.

Post-dive and after an individual correction of values due to the hematocrit variation, there is a reduction in the number of circulating platelets in all groups, without there being a significant difference in their raw value or the proportion of these variations between the different groups (Platelet count: KW_Ctrl/Flux/CtrlNoDivers/FluxNoDivers_, *n* = 19/20/4/4, *p* = 0.646; KW_Nodivers/SevereDCS/DCS/NoDCS_, *n* = 8/13/6/20, *p* = 0.766; Platelets%: KW_Ctrl/Flux/CtrlNoDivers/FluxNoDivers_, *n* = 19/20/4/4, *p* = 0.613; KW_Nodivers/SevereDCS/DCS/NoDCS_, *n* = 8/13/6/20, *p* = 0.678), which suggests that these variations are potentially due to the stress of the sample collection or the hyperbaric protocol (simulated or real).

There is a reduction in the number of circulating red cells only post-dive, without there being a significant difference in their raw value or the proportion of these variations between the different groups (RBC count: KW_Ctrl/Flux/CtrlNoDivers/FluxNoDivers_, *n* = 19/20/4/4, *p* = 0.657; KW_Nodivers/SevereDCS/DCS/NoDCS_, *n* = 8/13/6/20, *p* = 0.645; RBC%: KW_Ctrl/Flux/CtrlNoDivers/FluxNoDivers_, *n* = 19/20/4/4, *p* = 0.832; KW_Nodivers/SevereDCS/DCS/NoDCS_, *n* = 8/13/6/20, *p* = 0.550), which suggests that these variations are potentially due to the stress of the hyperbaric protocol (real). It is similar for hemoglobin.

A significant reduction in the number of circulating leukocytes is observed in the Ctrl but not in the Flux, without differences appearing in the No Diver rats. In proportion, the reduction in the number of circulating leukocytes is much less significant (KW_Ctrl/Flux/CtrlNoDivers/FluxNoDivers_, *n* = 19/20/4/4, *p* = 0.024) in the surviving Flux (2.78 ± 33.2%) than in the surviving Ctrl (−19.3 ± 23.7%) or the No Diver Flux. Factually, this variation cannot be attributed directly to the hemoconcentration, or to the stress caused by handling, but to the hyperbaric protocol (real) and the consequences of it on the one hand, and to the effect of fluoxetine on the other hand since there is no difference in proportion linked to the clinical status (KW_Nodivers/SevereDCS/DCS/NoDCS_, *n* = 8/13/6/20, *p* = 0.228).

When the clinical data is examined, the main post-dive rheological difference lies in the raw value of the hematocrit, which is higher in the Severe DCS compared to the NoDCS. When focus is placed on the effect of the treatment, fluoxetine seems to reduce the recruitment of circulating leukocytes after hyperbaric exposure.

### Blood biochemistry

Following the hyperbaric protocol the biochemistry was carried out using blood taken from the vena cava. No significant difference was observed between the two groups of non-exposed and NoDCS rats. It was similar when the NoDivers rats were compared between themselves (Ctrl and Flux), apart from the globulin which was higher in the Flux than the Ctrl (globulin NoDivers KW_Ctrl/Flux_; *n* = 4/4; *p* = 0.019) which is seen as a mild (pro)inflammatory syndrome or liver failure.

**Figure 6:**
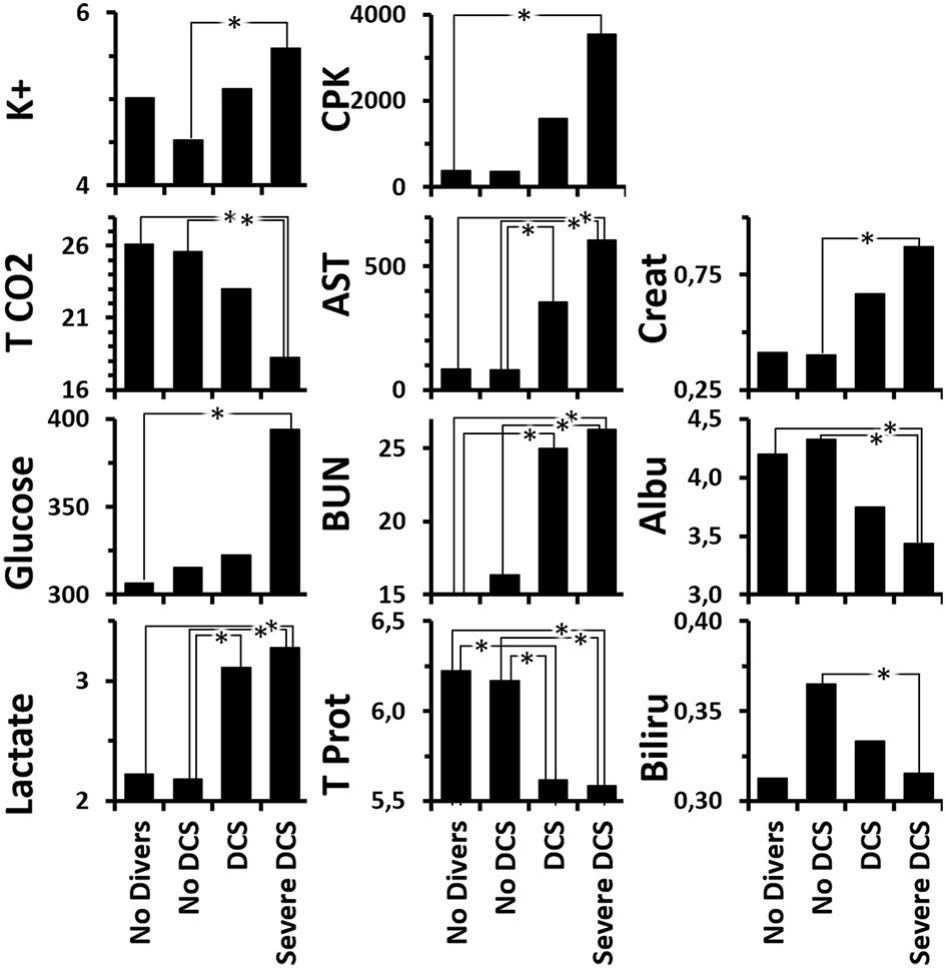
Analysis of blood biochemistry differences after the hyperbaric protocol according to the clinical status of rats. K+ (mmol/L), T CO_2_ (mmol/L), Glucose (mg/dL), Lactate (mmol/L), CPK/Creatinine Kinase (U/L), AST/Transaminase (U/L), BUN/UREA (mg/dL), Total Protein (g/dL), Creatinine (mg/dL), Albumin (g/dL), Bilirubin (mg/dL). ^*^Significant difference in *post-hoc* analysis (*p* < 0.05) (see Supplementary Table 2 for statistical details).

Globally (Figure 6 and Supplementary Table 2 for statistical analysis) the DCS rats present a low level of total proteins suggesting either hemodilution (but there is an increase in the hematocrit) or a protein leak due to a capillary leak or a renal lesion. These animals also present high levels of uric acid, transaminases and lactate, which taken together could be linked to hemoconcentration. Taken independently, the latter three elements suggest a defect in glomerular filtration, hepatic impairment and anaerobic functioning, respectively. As was expected, the SevereDCS presented irregularities for the same elements with, in addition, lower concentrations of albumin, creatinine, and globulin (a toxic, hydrophobic breakdown product of red blood cells that is bound to and carried by albumin), which accompanied the global reduction in the level of total proteins already recorded in the DCS statuses, confirming the capillary leak or glomerular lesion. A glomerular lesion could also explain the high levels of circulating urea. In addition, the high levels of transaminases lead to a suspicion of a liver disorder, possibly with the presence of bubbles in the liver. More seriously, the joint reduction in the TCO_2_ and the increase in the levels of glucose and lactate lead us to consider a defect in the respiratory chain accompanied by cell breakdown, explained by high levels of potassium and CPK (muscle or heart straining) on the other hand. It all suggests a multi-organ failure.

**Figure 7:**
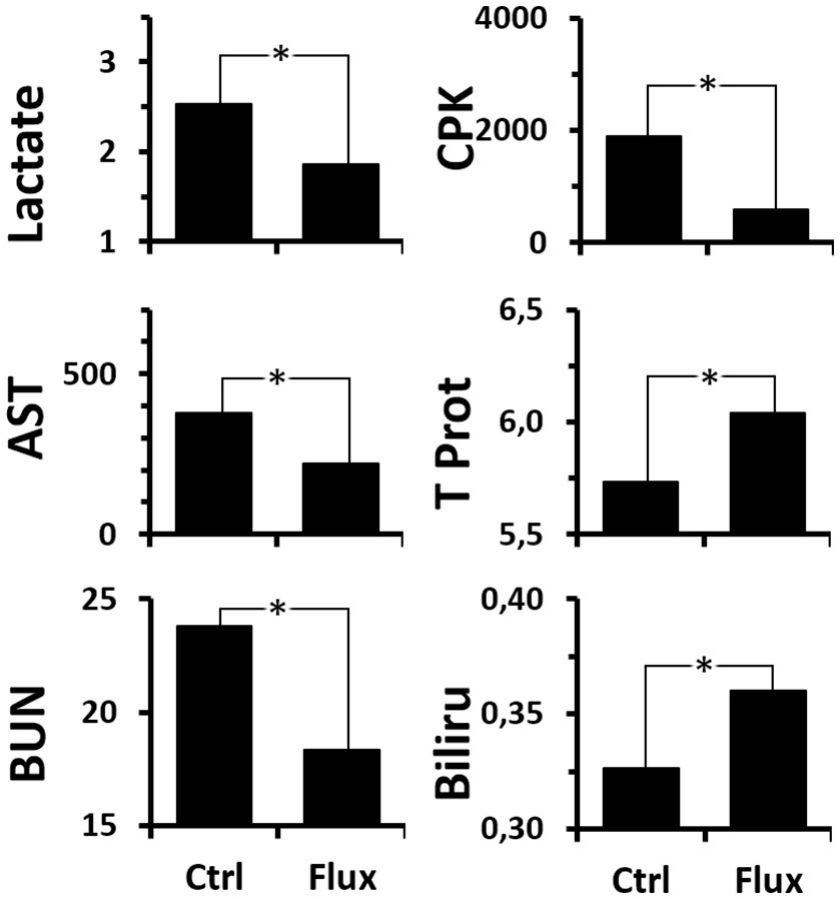
Analysis of blood biochemistry differences after the hyperbaric protocol according to rat treatments. Lactate (mmol/L), CPK/Creatinine Kinase (U/L), AST/Transaminase (U/L), BUN/UREA (mg/dL), Total Protein (g/dL), Bilirubin (mg/dL). ^*^Significant difference (*p* < 0.05) (see Supplementary Table 3 for statistical details).

When more particular interest is taken in the actual effect of the chronic treatment with fluoxetine on the survivors (Figure 7 and Supplementary Table 3 for statistical analysis), a global improvement in the biochemical profile is observed compared to the Ctrl. Initially it appeared that recourse to anaerobic respiration (production of lactate) is less pronounced. It also appears that the hepatic and renal malfunctions (transaminases and urea) associated with capillary leaks (Total Protein, Bilirubin) and, more generally, the muscle and cell stress (CPK) are less significant.

### Inflammatory cytokines

The non-exposed rats treated with fluoxetine tend to present lower levels of circulating IL-1 beta than animals which did not receive treatment, whether non-exposed or not (IL-1 beta: KW_Ctrl/Flux/CtrlNoDivers/FluxNoDivers_, *n* = 19/20/4/4, *p* = 0.091; _/_ *post-hoc*_CtrlNoDivers/FluxNoDivers_ *p* = 0.016;/*post-hoc*_Ctrl/FluxNoDivers_ *p* = 0.044).

### Circulating oligonucleotides

**Figure 8:**
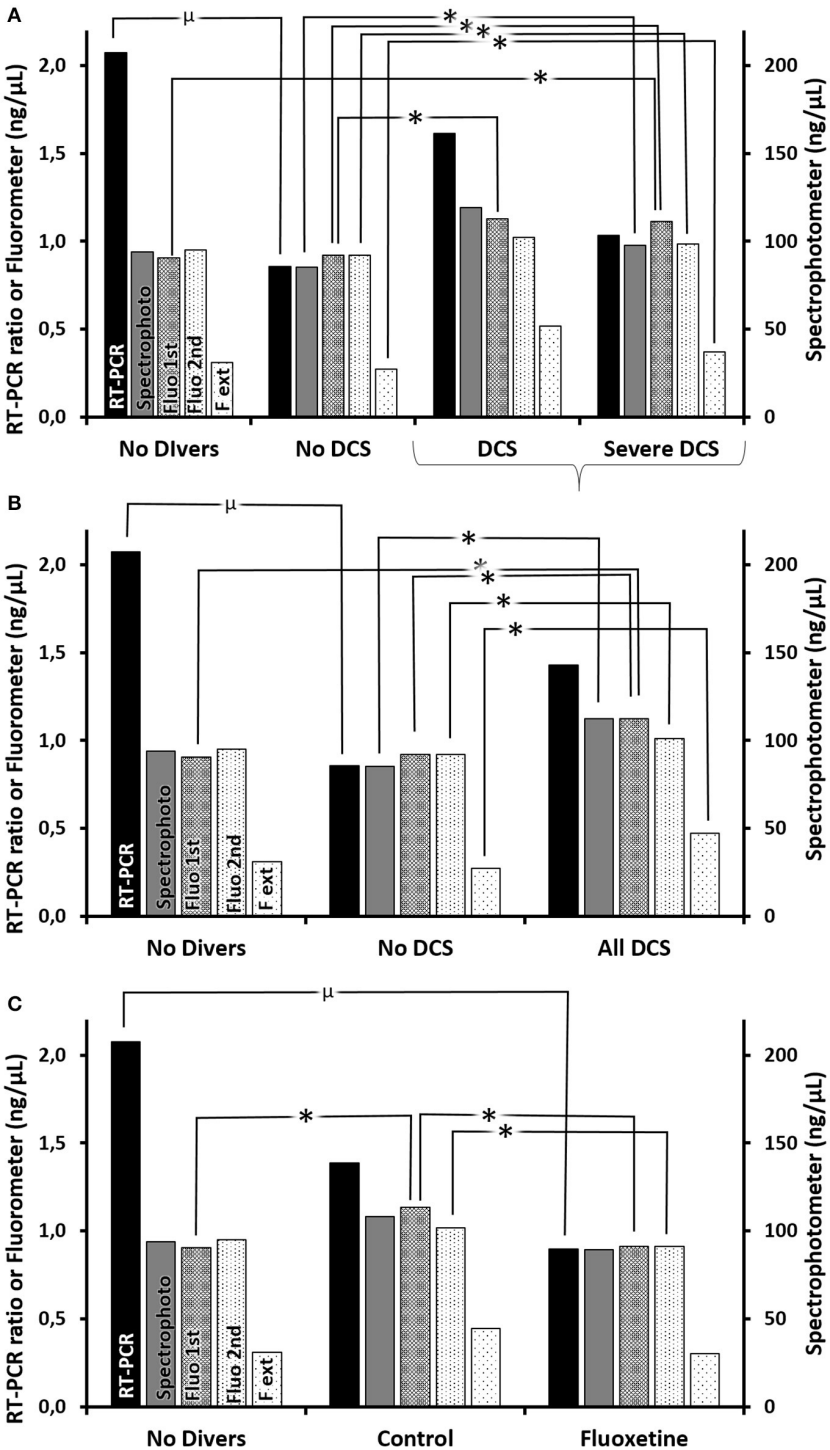
**(A–C)** Circulating oligonucleotides according to clinical status **(A)** with all DCS together **(B)** or according to treatment. **(C)** Quantifications of oligonucleotides were performed (1) by RT-PCR (black histograms) including double centrifuging before the extraction/purification phase, which was followed by a qPCR targeting mDNA and an internal standard (i.e., ratio), and which includes (2) (gray histograms) the measurement by spectrophotometry (nanodrop) post-extraction usually used to adjust the parameters of the qPCR, and (3) (histograms with dots) by direct fluorimetric measurement at the different stages (1st centrifuging → Fluo 1st, 2nd centrifugating → Fluo 2nd, extraction/amplification → F ext) targeting the double strands of DNA without distinction. ^*^Significant difference, or μ a trend, in *post-hoc* analysis (*p* < 0.05) (see Supplementary Tables 4–6 for statistical details).

In order to understand better the interest of the different assay methods, it was necessary to present the results according to two clinical classifications, the first technique (Figure 8A and Supplementary Table 4 for statistical analysis) being more restrictive than the second (Figure 8B and Supplementary Table 5) covering all the rats which had decompression sickness (All DCS = DCS + Severe DCS). So it appears generally that, amongst the animals exposed to the decompression protocol, the animals which experienced decompression sickness (SevereDCS, DCS, or All DCS) present higher circulating DNA values than animals that are unscathed (NoDCS) (Figures 8A,B and Supplementary Tables 4, 5), whatever the method envisaged.

However, it should be noted that there is a discordance in the DNA measurements between the measurements made with the Quantus fluorometer measuring the total double strand quantity (histograms with dots), which is expressed as the trend AllDCS>NoDCS>NoDivers, and those that target the mitochondrial DNA (black histograms) which tends to conclude that NoDivers>AllDCS>NoDCS (Figures 8A,B).

By taking a more particular interest in the effect of the treatment in the survivors (Figure 8C and Supplementary Table 6 for statistical analysis), it is generally noted that the Ctrl rats have higher levels of circulating oligonucleotides compared with rates having received fluoxetine, or No Diver rats.

## Discussion

Like previous studies the dive protocol has indeed caused cases of DCS (Pontier et al., 2008; Blatteau et al., 2012, 2015; Vallee et al., 2013; De Maistre et al., 2016), globally seen as an alteration in physical and behavioral performances accompanied by a deterioration of biological constants, all groups included. However, chronic treatment with fluoxetine modifies the rat performances both significantly and favorably during the physical and behavioral tests, just like their biological and biochemical constants, following exposure to the hazardous protocol. It does not, however, offer such as, beneficial effect as a high dose of fluoxetine of 50 mg/kg administered on an *ad hoc* basis (Blatteau et al., 2015).

Before the dive a mild effect from the chronic treatment with fluoxetine can be seen. Also, it appears that the Flux animals have an increased Mean Corpuscular Volume and a Mean Corpuscular Hemoglobin, as well as increased globulin levels. This resulted in a mild (pro) inflammatory syndrome (also in line with the increase in pro-inflammatory IL-1 betas) or mild liver failure. This mild inflammation could explain why the algesic sensitivity of the left hind paw was more pronounced in No Dive treated rats. In addition, this would explain their difficulty in moving on the narrow plank above the void (reduction in agility). For all that, their exploratory behavior was not affected when it involved moving in a less restrictive area (flat surface). However, this could also be due to a sampling effect.

Following the hyperbaric protocol and from a clinical point of view, the rats presenting DCS (whether Ctrl or Flux) had inhibited exploratory behavior and reduced motor and locomotor scores for the beam test. Nevertheless, this exploratory behavior was significantly less degraded in rats treated with fluoxetine, which means that the rats were either less stressed as a result of (a removal of inhibition by) their anti-depressant treatment or suffered less damage than the Ctrl rats and therefore were more able to move about. This corresponds to the description by Kaur and Kulkarni (2002), which describes fluoxetine as limiting the immobility induced by hyperalgesia. This is comparable to the previous study by Blatteau et al. (2015) even though the fluoxetine was administered acutely. This result is interesting because a loss of sensitivity (nociception) is regularly observed in humans after decompression sickness, and is found again in the P DCS rats.

Generally and rheologically, the protocol producing DCS has increased the hematocrit and significantly reduced the number of circulating erythrocytes and leukocytes, as well as the number of circulating platelets. This is generally attributed to prothrombotic phenomena and an inflammation causing diapedesis. However, the reduction in the number of leukocytes was significantly lower in the Flux rats, which implies that fluoxetine would reduce diapedesis, as has been observed in previous studies (Blatteau et al., 2012, 2015; Vallee et al., 2016). These effects are generally attributed either to the direct interaction of bubbles circulating with the platelets, or to the abrasion of the endothelial cells by these circulating bubbles (Nossum et al., 1999, 2002). In parallel, the question is whether there is a pathological hematocrit value as described in this work with the Severe DCS group. This situation was previously described in the work of Musallam et al. (2013) where the effect on mortality was noted beyond the hematocrit thresholds of 0.48 in women and 0.52 in men, and the effect were considerably higher for values exceeding 0.54.

The biochemical analyses show a capillary leak, a symptom observed in humans in critical cases, which would explain the recorded hemoconcentration (Gempp et al., 2013, 2014).

The stress of decompression is felt most particularly in the cells via the measurement of circulating oligonucleotides, which is more significant in the rats with decompression sickness. Fluoxetine again seems to improve this constant whatever the DNA measurement considered. The increase in circulating oligonucleotides was initially attributed to the destruction of cells by bubble abrasion following necrotic phenomena (Vallee et al., 2012). When considering total double strand DNA it is seen that Divers without accidents have more circulating DNA than No Divers, which reinforces the previous theory and also suggests that the presence of bubbles is possible and that it can break down some cells without necessarily being pathological for the organism. In humans, we sometimes talk about “Bubble-resistant Subjects,” referring to individuals in whom high levels of circulating bubbles are detected but who do not develop symptoms. In a contradictory way, this work also shows that the Divers without accidents had less mitochondrial DNA (this time) than No Divers, which suggests that this DNA is specifically broken down by the hyperbaric exposure. Given that the other technique for DNA detection (total double strand) is less specific than a sequence, the only hypothesis that we could put forward at this stage would be premature mitochondrial death linked to oxidative stress (see Shokolenko et al., 2009; Resseguie et al., 2015) causing the breakage of amplified sequences linked to the hyperbaric exposure and their high oxygen contents. In particular, this hypothesis relies on the over-production of ROS (Reactive Oxygen Species) and HSP (Heat Shock Protein) in a dive (Eftedal et al., 2013) and the impact of these components on the integrity of the DNA strands (Shokolenko et al., 2009; Hobani, 2016; Rima et al., 2016), as well as the work of Cacciuttolo et al. (1993) and Witte et al. (2014) recounting a direct effect of hyperoxia on the breakdown of DNA strands in the cells of mammals. In fact, the oxidative stress targets the genome guardians and particularly the repeated sequences rich in guanine, particularly telomeres (Thilagavathi et al., 2013a,b; Mishra et al., 2016). This result therefore raises the question of the toxicity of hyperoxic hyperbaric and normobaric exposures in humans (see O'reilly, 2001), whether therapeutic or not.

The acute dose of fluoxetine (50 mg/kg), as opposed to the lower chronic dose of 20 mg/kg, delivers better neuroprotection against decompression sickness when the TREK 1 channels have previously been blocked by spadin (Vallee et al., 2016): they inhibit NMDA-R receptors, regulate the inflammatory effects, and have analgesic properties. This same acute dose of 50 mg/kg administered alone seems to have less effect (Blatteau et al., 2012, 2015). However, it seems more advantageous than the chronic dose used in this study, with them all remaining effective. This would confirm a major, dose-dependent effect of fluoxetine potentializing its anti-inflammatory effect.

It could be concluded that the Ctrl DCS rats face a clinical assessment tending toward multi-organ failure in the most severe cases, which is countered by fluoxetine in Flux rats. The presence of disseminated coagulation and cell destruction in the widest sense justifies the initial appearance of bubbles (DCS), which causes a progressive inflammatory syndrome, which defines decompression sickness. The beneficial effect of fluoxetine is noted here. It predicts a favorable clinical prognosis for DCS and would compensate for the loss of neuroprotection due to the inhibition of TREK 1, as it has been able to be shown in previous works (Vallee et al., 2012; Blatteau et al., 2015).

## Conclusion

Apart from the psychic effects, fluoxetine taken for 35 days does not have a negative incidence on the rate of DCS in the rat model. On the contrary, it improves the results of behavioral tests and the rheological and biochemical results, following the protocol producing decompression sickness. The effects of chronic treatment with fluoxetine are similar to those for an acute dose, but they seem to be dose-dependent. The fact that fluoxetine is unlikely to exacerbate the pathology associated with decompression would not allow the inclusion of treatment with fluoxetine, and possibly and more generally treatments with SSRIs, in the list of contraindications for scuba diving. This will add weight to further investigations on humans particularly epidemiological.

## Author contributions

JB and NV conception and design of research; CC, SD, and NV performed experiments; CC and NV analyzed data; CC, JB, SD, JA, LC, JR, and NV interpreted results of experiments; NV prepared figures; CC and NV drafted, edited, and revised manuscript; CC, SD, JA, LC, JB, JR, and NV approved final version of manuscript.

### Conflict of interest statement

The authors declare that the research was conducted in the absence of any commercial or financial relationships that could be construed as a potential conflict of interest.
