# Supplementary material for: Thirty-five Day Fluoxetine Treatment Limits Sensory-Motor Deficit and Biochemical Disorders in a Rat Model of Decompression Sickness
Source: Front Physiol. 2017 Sep 5;8:604. doi: 10.3389/fphys.2017.00604 (PMC5591790; doi:10.3389/fphys.2017.00604)
Supplement: Supplementary file 1 [file DataSheet2.DOCX]

**Criteria of human end point**

| **degree of constraint from 0 (no constraint) to 3 (high stress)** | | | | |
| --- | --- | --- | --- | --- |
| Date : | Group : | Protocole : | | |
|  | Rat X | Rat Y | Rat Z | Rat… |
| **Behavior** |  | | | |
| Vocalize (shouting, crying) |  |  |  |  |
| withdrawn behavior |  |  |  |  |
| Agression behavior |  |  |  |  |
| Reduct° in exploratory behavior |  |  |  |  |
| Licking |  |  |  |  |
| Isolation from the group |  |  |  |  |
| **Eyes** |  | | | |
| Closed eyeslids |  |  |  |  |
| Sunken eyes |  |  |  |  |
| Tears |  |  |  |  |
| Bubles in the eyes |  |  |  |  |
| **Breath** |  | | | |
| High respiratory rate |  |  |  |  |
| Labored breathing |  |  |  |  |
| Gasps |  |  |  |  |
| Runny nose |  |  |  |  |
| **Appearance** |  | | | |
| Fur bristling |  |  |  |  |
| Arched back |  |  |  |  |
| Purple skin (hypoxya) |  |  |  |  |
| **Locomotion** |  | | | |
| Inhibited locomotion |  |  |  |  |
| Limping |  |  |  |  |
| Staggering gait |  |  |  |  |
| Lying down |  |  |  |  |
| Lying on its side |  |  |  |  |
| **Fore limbs** |  | | | |
| Atony |  |  |  |  |
| Paresis |  |  |  |  |
| Paralysis |  |  |  |  |
| **Right hind paw** |  | | | |
| Atony |  |  |  |  |
| Paresis |  |  |  |  |
| Paralysis |  |  |  |  |
| **Left hind paw** |  | | | |
| Atony |  |  |  |  |
| Paresis |  |  |  |  |
| Paralysis |  |  |  |  |
| **Convulsion** |  |  |  |  |
| **Pain during anesthesia** |  |  |  |  |
| **TOTAL SCORE:** |  |  |  |  |

According to our animal care committee, we used a system to determine when
the experiment must be stop, during the observation period. In all cases,
animals were continuously observed for 30 minutes after the end of the dive.

The table is inspired by the swiss veterinary guideline. The dedicated observer completes the form, given that a pain of degree 3 (very painful) in one case or a total amount of 12 are a stop point.

If sacrifice is needed, animal is first anesthetized with halothane (5% in
oxygen, Halothane, Belamont, France) and then sacrificed by injecting pentobarbital (200 mg/kg ip, Sanofi Santée, France). The gazeous anesthesia (halothane) is used in order to gain
time and to avoid to add another stress.

| Effect of clinical status on: | KW_NoDivers/SevereDCS/DCS/NoDCS_  p-value for n=8/13/6/20 | Significant *post-hoc* test (p<0.0083) | |
| --- | --- | --- | --- |
| K+ | 0.004 | SevereDCS > NoDCS | p = 0.0003 |
| T CO2 | <0.0001 | SevereDCS < NoDCS  SevereDCS < NoDivers | p < 0.0001  p < 0.0001 |
| Glucose | 0.015 | SevereDCS > NoDCS | p = 0.0031 |
| Lactate | <0.0001 | SevereDCS > NoDCS  SevereDCS > NoDivers  DCS > NoDCS | p = 0.0001  p =0.0031  p = 0.0026 |
| Creatinine Kinase | 0.004 | SevereDCS > NoDCS | p = 0.0083 |
| AST/Transaminase | <0.0001 | SevereDCS > NoDCS  SevereDCS > NoDivers  DCS > NoDCS | p < 0.0001  p = 0.0022  p = 0.0010 |
| BUN/Urea | <0.0001 | SevereDCS > NoDCS  SevereDCS > NoDivers  DCS > NoDivers | p = 0.0001  p = 0.0003  p = 0.0055 |
| Total protein | <0.0001 | SevereDCS < NoDCS  SevereDCS < NoDivers  DCS < NoDCS  DCS < NoDivers | p < 0.0001  p = 0.0001  p = 0.0021  p = 0.0020 |
| Creatinine | 0.003 | SevereDCS < NoDCS | p = 0.0004 |
| Albumin | <0.0001 | SevereDCS < NoDCS  SevereDCS < NoDivers | p < 0.0001  p = 0.0025 |
| Bilirubin | 0.012 | SevereDCS < NoDCS | p = 0.0045 |

**Table 2 : Analysis of blood biochemistry differences after the hyperbaric protocol according to the clinical status of rats.**

| Effect of treatment post-dive on: | MW_Ctrl/Flux_  p-value for n=19/20 | Significance |
| --- | --- | --- |
| Lactate | 0.009 | Ctrl > Flux |
| Creatinine Kinase | 0.017 | Ctrl > Flux |
| AST/Transaminase | 0.049 | Ctrl > Flux |
| BUN/Urea | 0.050 | Ctrl > Flux |
| Total protein | 0.021 | Ctrl < Flux |
| Bilirubin | 0.038 | Ctrl < Flux |

**Table 3 : Analysis of blood biochemistry differences after the hyperbaric protocol according to rat treatments**

| circulating oligonucleotides  according to clinical status, after: | KW_NoDivers/SevereDCS/DCS/NoDCS_  p-value for n=8/13/6/20 | p-value of post-hoc test  (significant for p<0.0083) | |
| --- | --- | --- | --- |
| Fluorometer after 1^st^ centrifugation | 0.001 | SevereDCS > NoDCS  SevereDCS > NoDivers  DCS > NoDCS | 0.001  0.011  0.009 |
| Fluorometer after 2^nd^ centrifugation | *0.050* | SevereDCS > NoDCS | 0.007 |
| Fluorometer after extraction | 0.003 | SevereDCS > NoDCS | 0.000 |
| Spectrophotometer after extraction | *0.056* | SevereDCS > NoDCS | 0.007 |
| RT-PCR Mito/SPC ratio | *0.055* | NoDivers > NoDCS | *0.015* |
| *Italic typography marks a trend* | | | |

**Table 4 Circulating oligonucleotides levels according to clinical status.**

| circulating oligonucleotides according to clinical status, with all DCS together, after: | KW_NoDivers/AllDCS/NoDCS_  p-value for n=8/19/20 | p-value of post-hoc test  (significant for p<0.0083) | |
| --- | --- | --- | --- |
| Fluorometer after 1^st^ centrifugation | 0.000 | All DCS > NoDCS  All DCS > NoDivers | 0.000  0.007 |
| Fluorometer after 2^nd^ centrifugation | 0.024 | All DCS > NoDCS | 0.006 |
| Fluorometer after extraction | 0.001 | All DCS > NoDCS | 0.000 |
| Spectrophotometer after extraction | 0.027 | All DCS > NoDCS | 0.007 |
| RT-PCR Mito/SPC ratio | 0.028 | NoDivers > NoDCS | *0.015* |
| *Italic typography marks a trend* | | | |

**Table 5 Circulating oligonucleotides levels according to clinical status, with all DCS together.**

| **Effect of treatment on**  **circulating oligonucleotides, after :** | **KW_NoDivers/Ctrl/Flux_**  p-value for n=8/19/20 | **p-value of post-hoc test**  (significant for p<0.0167) | |
| --- | --- | --- | --- |
| **Fluorometer after 1^st^ centrifugation** | 0.001 | Ctrl > Flux  Ctrl > NoDivers | 0.000  0.009 |
| **Fluorometer after 2^nd^ centrifugation** | 0.002 | Ctrl > Flux | 0.000 |
| **Fluorometer after extraction** | NS | NS |  |
| **Spectrophotometer after extraction** | NS | NS |  |
| **RT-PCR Mito/SPC ratio** | 0.035 | NoDivers > Flux | *0.017* |
| NS for non significant and, italic typography marks a trend | | | |

**Table 6 Circulating oligonucleotides levels according to the treatment.**
